# Quantitative Assessments of Mechanical Responses upon Radial Extracorporeal Shock Wave Therapy

**DOI:** 10.1002/advs.201700797

**Published:** 2017-12-19

**Authors:** Yajun Liu, Xiaodong Chen, Anyi Guo, Sijin Liu, Guoqing Hu

**Affiliations:** ^1^ Orthopedic Shock Wave Treatment Center Spine Surgery Department Beijing Jishuitan Hospital Beijing 100035 China; ^2^ The State Key Laboratory of Nonlinear Mechanics Beijing Key Laboratory of Engineered Construction and Mechanobiology Institute of Mechanics Chinese Academy of Sciences Beijing 100190 China; ^3^ School of Engineering Science University of Chinese Academy of Sciences Beijing 100049 China; ^4^ The State Key Laboratory of Environmental Chemistry and Ecotoxicology Research Center for Eco‐Environmental Sciences Chinese Academy of Sciences Beijing 100085 China

**Keywords:** experimental measurements, explicit dynamics analysis, extracorporeal shock wave therapy, finite element models

## Abstract

Although radial extracorporeal shock wave therapy (rESWT) has been widely used to treat orthopedic disorders with promising clinical results, rESWT largely relies on clinicians' personal experiences and arbitrary judgments, without knowing relationships between administration doses and effective doses at target sites. In fact, practitioners lack a general and reliable way to assess propagation and distribution of pressure waves inside biological tissues quantitatively. This study develops a methodology to combine experimental measurements and computational simulations to obtain pressure fields from rESWT through calibrating and validating computational models with experimental measurements. Wave pressures at the bottom of a petri dish and inside biological tissues are measured, respectively, by attaching and implanting flexible membrane sensors. Detailed wave dynamics are simulated through explicit finite element analyses. The data decipher that waves from rESWT radiate directionally and can be modeled as acoustic waves generated from a vibrating circular piston. Models are thus established to correlate pressure amplitudes at the bottom of petri dishes and in the axial direction of biological tissues. Additionally, a pilot simulation upon rESWT for human lumbar reveals a detailed and realistic pressure field mapping. This study will open a new avenue of personalized treatment planning and mechanism research for rESWT.

## 1 Introduction

As a noninvasive technology, extracorporeal shock wave therapy (ESWT), was developed from the extracorporeal shock wave lithotripsy that is conventionally used to destroy kidney stones or biliary calculi using an externally applied, focused, and high‐intensity acoustic pulse.^1^ Further, ESWT was also customized to treat orthopedic diseases, for example, pseudarthrosis,^2^ plantar fasciitis,^3^ lateral epicondylitis,^4^ shoulder calcific tendinitis,^5^ Achilles tendinopathy,^6^ and osteonecrosis of the femoral head.^7^ There are two types of ESWT according to their wave patterns, namely, focused and radial. By definitions, focused ESWT creates a pressure field with a focal zone in the treatment region, while radial ESWT (rESWT) generates radially expanding pressure. In the rESWT device, the projectile is driven by compressed air or a magnetic field to impact onto the applicator at the head end of a handpiece for wave generation. As an economic and noninvasive physical therapy with good clinical effects, applications of rESWT have gradually increased in recent years.^8^

Different hypotheses have been proposed to explain the biological effects of ESWT, e.g., cavitation‐induced microinjury promoting neovascularization,^9^ biological response at the cellular level,^10^ and pain management through the reduction of afferent sensory fiber function.^11^ Nonetheless, the actual mechanisms for the treatment of musculoskeletal disorders still remain a puzzle to be solved.^12^ There is no available relationship between any known mechanical quantity and a specific biological effect. Currently, rESWT largely relies on clinicians' personal experiences and arbitrary judgments, without knowing relationships between the applied dose (in terms of settings of devices) and a specific therapeutic dose at a target site (or “dose to tissue”) for different therapies or individuals, as well as biological response at target sites. Biological and medical effects cannot be predicted to establish patient selection criteria and optimal treatment parameters for musculoskeletal problems. Looking to radiotherapy as a model, a potential method to establish dose–response relationship is to define the term “effective dose,” which accounts for the relative sensitivities of different types of tissue using tissue weighting factors.^13^ Similar to therapeutic applications of ultrasound,^14^ it will require substantial researches to determine appropriate weighting factors by selecting and collecting appropriate clinical trials for rESWT, but first requirement is to know the pressure information at the target site, i.e., dose to tissue. Measuring the biological response without being able to measure the mechanical stimulus at a target site does not allow prediction of response.^15^

The characterization of wave propagations in biological tissues is a prerequisite to decipher the mechanism underlying the biological and medical effects. To date, experiments within water have been used to characterize acoustic fields of rESWT devices since direct measurements in tissues are impossible to acquire in most cases.[[qv: 12b,16]] Silicon pads were also used to propose a “dry test” bench using an accelerometer for the characterization of the rESW.^17^ Nevertheless, only several studies indeed measured the pressure inside biological tissues. For example, Benoit et al. measured the pressure wave propagation in the samples of porcine skin and adipose by a pressure gage placed behind the tissue and backed by an impedance‐matched foil and silicon rubber.^18^ Combining with numerical simulations, their results highlighted the nonlinear viscoelastic effects of biological tissues on characterizing wave propagations. Recently, Cirovic et al. determined the pressure generated by rESWT at the deep surface of the plantar fascia in human cadaveric feet by carefully inserting a needle hydrophone.^19^ Finite element models of rESWT were also established by the same group to simulate the rESWT treatments for chronic plantar fasciitis.^20^ Along with the clinic observations, preliminary cellular experiments in petri dishes have been used to study the cellular‐level biological responses to rESWT.^21^ However, it is questionable whether rESWT applied in cellular experiments in petri dish could realistically reflect the real situation inside human body. To this end, to avoid placebo effects or poor treatments, quantitative assessments of rESW's pressure at the target sites are thus essential to provide the basics of both clinical and laboratory investigations.

In this study, we established a methodology through combining experimental measurement and computational modeling to provide detailed information about pressure waves generated from rESWT. By attaching and implanting flexible membrane sensors, wave pressures at the bottom of petri dishes and inside porcine tissues were determined, respectively. Computational models were constructed to simulate wave propagations in both water and biological tissues using an explicit dynamics method. Quantitative assessments of wave pressures were ensured by calibrating and validating numerical results and parameters with experimental measurements. Detailed 3D wave propagations and distributions in the petri dishes and biological tissues were obtained to reveal a radial propagation with directional distribution of rESW. Simple models were proposed to correlate the pressure distribution and guide clinical applications. The influence of wave reflection was also analyzed to draw attentions to situations involving large variations of acoustic impedances across material interfaces. Our methodology with calibration and validation may likely find widespread applications in in vitro and in vivo studies of rESWT. Our combined methodology could be used to build the link between applied dose (in terms of settings of devices) and dose to tissue for different therapies or individuals. Coupling with the dose–response relationship for different therapies obtained from clinic trials, the amount of applied doses can be determined to make therapies safer and more effective.

## 2 Results and Discussion

### 2.1 Pressure Field in Petri Dish

**Figure 1:**
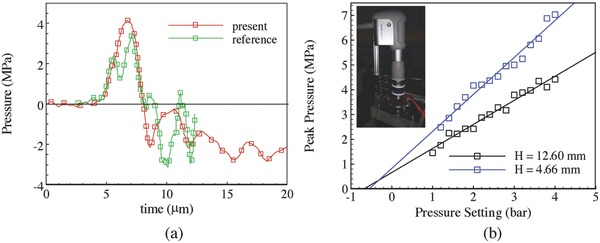
a) Pressure waveform at the bottom of a petri dish compared to the measurement in a free field[[qv: 17b]] for driving pressure of 2 bar. b) Variations of the maximum positive pressure with the device driving pressures for two depths.

The pressure evolutions at the bottom center of a petri dish were measured inside water for two depths *H*, which is the vertical distance between the applicator and the pressure sensor. The driving pressure from the rESWT device *P*_in_ varied from 1 to 4 bar since *P*_in_ more than 4 bar has shown not to increase the pressure amplitudes any further.[[qv: 17b]] All measurements were performed at the lowest driving frequency of 1 Hz. Note that previous measurements showed that the positive pressure amplitude decreased about 27.1% when the driven frequency increased from 1 to 10 Hz.[[qv: 17b]] **Figure** **1**a shows the measurement for *P*_in_ = 2 bar and *H* = 4.66 mm. The results were compared with the free field measurement by a hydrophone for a slightly larger measure distance (*H* = 5 mm).[[qv: 17b]] Both the duration and the amplitude of the first positive pulse were consistent with experimental results.[[qv: 17b]] Linear variations of the maximum positive pressure of the first pulse *P*_+_ with *P*_in_ for the two depths *H* were observed in Figure 1b. As expected, the variation for *H* = 4.66 mm was in agreement with previous measurements for *H* = 5 mm.[[qv: 17b]] Note that small bubbles were observed for all *P*_in_, indicative of the occurrences of cavitation in water.[[qv: 16b,22]]

Cellular‐level understanding of the effect of pressure wave usually relies on in vitro cellular culture experiments. Our pressure measurements at the bottom of the petri dish were used directly to guide our cellular culture experiments that are in progress. However, it is difficult to experimentally obtain the spatial evolutions of the pressure wave. Numerical simulations were carried out to understand the pressure evolution inside the petri dish. Three simulations for grid independence study were carried out first using grid size Δ of 0.5, 1.0, and 2.0 mm. All the components were meshed with Δ elements except for the o‐rings, which were meshed with the finer Δ/2 elements to preserve their shapes. Figure S1 (Supporting Information) shows pressure evolutions at the bottom center for the three grid resolutions for *H* = 5.0 mm and *U* = 10.0 m s^−1^. It was found that the evolution of the wave pressure converges with the mesh. Balancing the result accuracy and the computing cost, Δ = 1.0 mm was used in the models of rESWT device, water and porcine tissues, while Δ = 2.0 mm was used in the model of human tissues. The total element numbers of the water, porcine, and human models were 285 362, 621 084, and 2 042 903, respectively.

**Figure 2:**
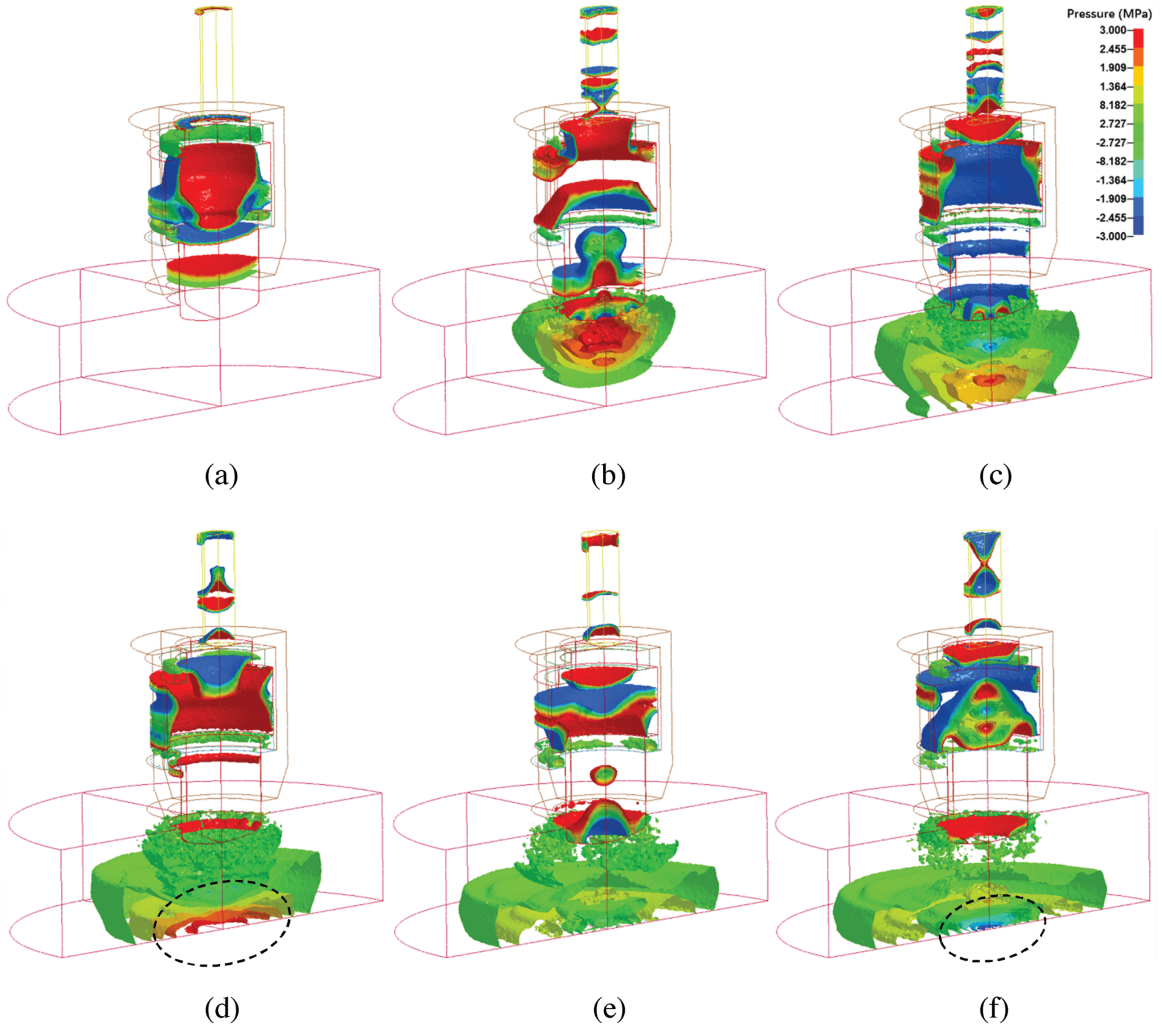
3D temporal and spatial distribution of the wave pressure in a petri dish for *H* = 12.6 mm and *U* = 10 m s^−1^. a) Pressure waves were generated inside the applicator by the impact of the projectile. b) Wave transmitted into water from the head end of the applicator to generate a pressure wave in water. c) The wave reached the bottom of the petri dish to result in a pressure gradient. d) The maximum pressure in the high‐pressure region (indicated by a dashed circle) was enhanced by the impeding of the bottom wall. e) The direction of the pressure gradient changed near the center. f) The negative pressure was also enhanced at the bottom wall (indicated by a dashed circle).

**Figure** **2** shows a representative propagation process of the pressure wave by isosurfaces for *H* = 12.6 mm. After the projectile collides the applicator, pressure waves formed inside the applicator to generate complex pressure oscillation inside the projectile and the applicator (Figure 2a). The propagation and reflection of the pressure pulse inside the applicator depended on geometry and material of the applicator.[[qv: 17b]] Transmitting from the head end of the applicator into water, the wavefront formed a mushroom‐like distribution of pressure isosurfaces (Figure 2b). As the wave propagated, the area of high‐pressure region was reduced due to the radial expansion of the wave. Once reaching the bottom of the petri dish the pressure distribution became flat (Figure 2c). Meanwhile, a pressure gradient was observed at the bottom. Remarkably, the maximum pressure in the high‐pressure region was enhanced as denoted by the dashed circle in Figure 2d. Since the bottom was fixed in the simulation, the velocity at the bottom was zero to result in an acoustically rigid boundary. The wave was reflected with no change in both amplitude and phase to result in a pressure amplitude twice that of the incident wave.^23^ The reflection at the bottom of the petri dish should then be carefully considered in cellular experiments, although in practice some cells may be exposed to reflected waves whereas others will not, depending on the position of the cells within the tissue.^24^ Figure 2e shows that the direction of the pressure gradient changes as the pressure at the dish center reduces. The negative pressure was also enhanced at the bottom wall as observed in Figure 2f.

**Figure 3:**
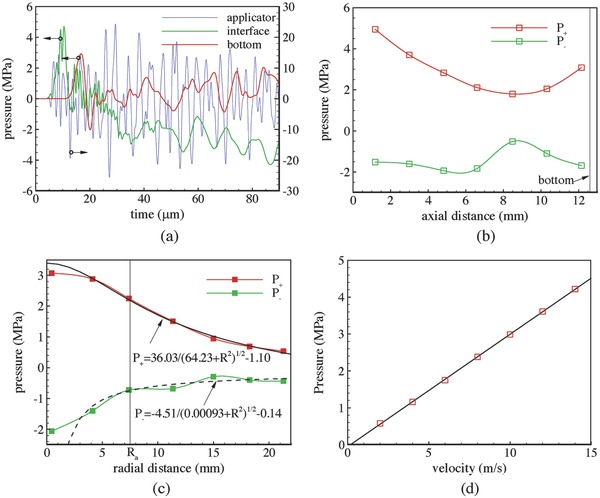
a) Typical pressure waveform at different axial positions for *U* = 10 m s^−1^. b) Axial distributions of *P*_+_ and *P*_−_. c) Radial distributions of *P*_+_ and *P*_−_ on the dish bottom. d) Variation of *P*_+_ at the dish bottom with *U* for *H* = 12.6 mm.

**Figure** **3**a depicts the variation of the waveform from the applicator to the bottom of the petri dish. At the tip of the applicator, the wave had an amplitude of about 10 MPa and a dominating frequency of around 0.3 MHz. In the water near the interface between the applicator and water, the wave frequency was in the order of 0.2 MHz with superimposed small waves with a frequency of about 0.7 MHz at the beginning. A positive wave pulse with about 8 µs duration was observed at the bottom, followed by a negative pulse with about 3 µs duration.

We found that the radiation of the wave pressure in Figure 2 was similar to that of a cone loudspeaker with a medium frequency, in which a vibrating plane piston set in a large baffle. Figure S2 (Supporting Information) shows a schematic diagram of a vibrating circular piston and a typical pressure contour. The far‐field pressure radiated by a baffled circular piston can be derived according to Rayleigh integral.^25^ If the plane piston with radius *r_p_* vibrates at a normal velocity of *u_ω_e^−iωt^*, the pressure amplitude is given by

(1) $$p_{\omega }(r)\;=\;-ik\rho c_{s}\frac{e^{ikr}}{4\pi r}f_{\omega }(\theta )\;and\;f_{\omega }(\theta )\;=\;2u_{\omega }\pi r_{p}^{2}\left[\frac{2J_{1}(kr_{p}sin\theta )}{kr_{p}sin\theta }\right]$$

where ρ is the density, *c*_s_ is the sound speed, *k* = ω/*c* is the wave number, *r* is the distance between the center of the piston to the observation point, θ is the direction angle with respect to the normal direction of the piston, and *J*_1_() is the first‐order Bessel function. The equivalent strength in direction θ, *f_ω_*(θ), is equal to twice the total outflow amplitude *u_ω_πr_p_* ^2^ times the directional factor in brackets, which is 1 at θ = 0. The directional factor shows that the distribution pattern depends on the value of *kr_p_*. If *kr_p_* < 1, the pressure radiates uniformly in all directions from the piston. If *kr_p_* > 1, the pressure radiates directionally to spread most energy within a cone around the piston axis. The angle of the cone is about sin^−1^(π/*kr_p_*).^25^ We found that, from Figure 3a, the period of the first wave generated from the rESWT device was about 11 µs. The value of *kr_p_* was about 3.23, indicating a directional radiation of rESW. Theoretically, the angle of the cone that contained most wave energy was about 76.5°, which agreed with the observation from simulations. The pressure field of rESW can thus be regarded to be generated from a circular piston with directional propagation.

Figure 3b shows the variations of *P*_+_ and *P*_−_ in the axial direction. Equation (1) indicates that pressure amplitudes are approximately proportional to 1/*r* along the axial direction. However, due to the impeding of the bottom wall, *P*_+_ decreased and then increased with the axial location. The absolute value of *P*_−_ also decreased and then increased near the bottom wall. Figure 3c shows *P*_+_ and *P*_−_ at points in different radial positions *R* on the bottom. To avoid numerical complications due to Bessel functions, we simply assumed that the directional factor is 1 at the bottom. Since pressure amplitudes are approximately proportional to 1/*r*, the pressure distribution at radial position *R* on the bottom can be expressed as

(2) $$P_{+/-}=a/\sqrt{{\left(H\;+\;b\right)}^{2}\;+\;R^{2}}\;+\;c$$

with three fitting parameters *a*, *b*, and *c*. The fitted curves in Figure 3c demonstrated that this simple equation described the pressure distribution on the bottom reasonably well.

The simulations were carried out for different impact velocity *U*. Figure 3d shows a linear relation between *P*_+_ and *U* at the bottom center. This linear variation was consistent with our experimental measurements in Figure 1b for *P*_in_ varying from 1 to 4 bar. Combining previous and our experimental measurements, the relationship between *P*_in_ and *U* can be calibrated. Values of *U* corresponding to *P*_in_ = 1, 2, 3, and 4 bar were 7.11, 12.46, 17.81, and 23.16 m s^−1^, respectively. Those values were used in latter sections to offer a direct comparison between our simulation and experiment results for porcine tissues.

### 2.2 Pressure Field in Porcine Tissues

**Figure 4:**
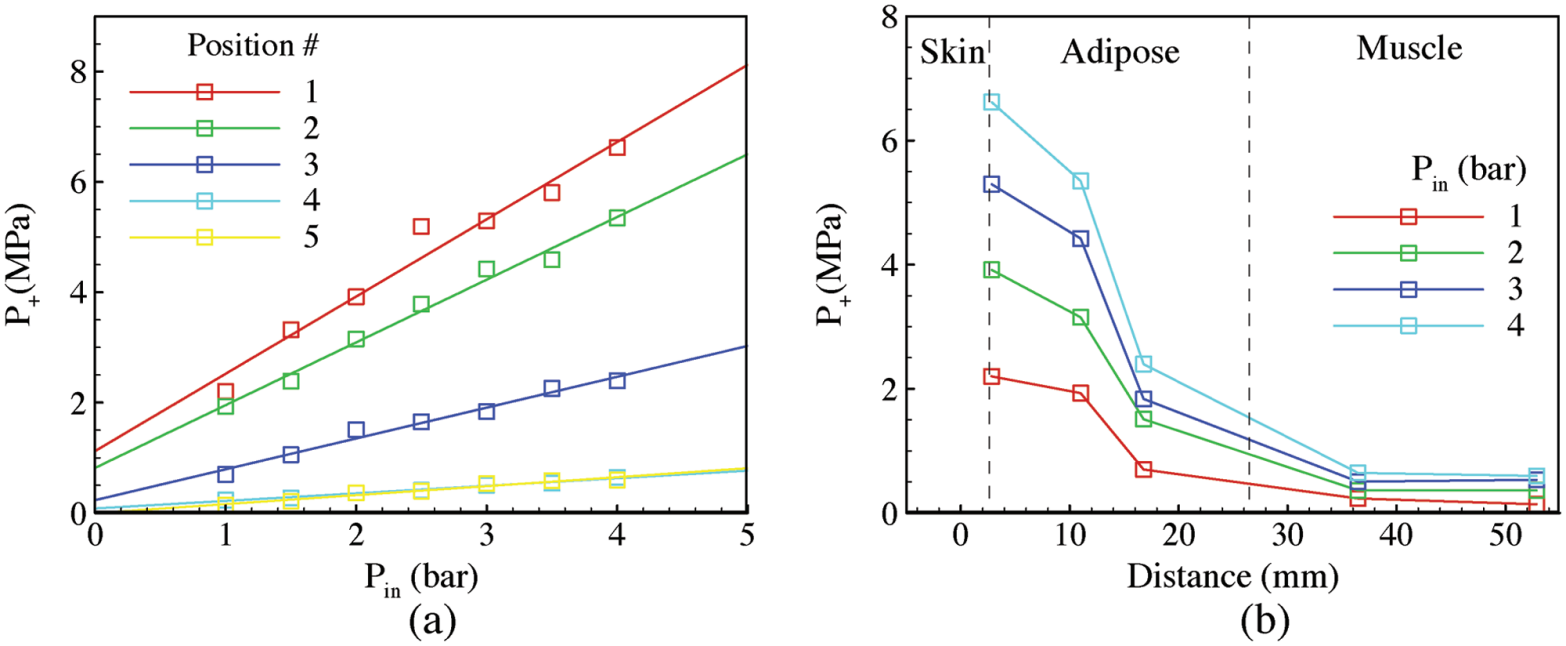
Measurements of *P*_+_ in porcine tissues. a) Variations of *P*_+_ with *P*_in_ for different *H*, 2.82, 11.00, 16.78, 36.46, and 52.86 mm. The thickness of skin and adipose tissues was 2.82 and 26.50 mm, respectively (detailed tissue thicknesses at each measured locations are listed in Table S1 of the Supporting Information). b) Variations of *P*_+_ with *H* for different *P*_in_.

The pressure inside porcine tissues was measured at five depths *H* varying from 2.82 to 52.86 mm for *P*_in_ = 1, 2, 3, 4 bar. As shown in Table S1 (Supporting Information), the depths covered three types of soft tissues—skin, adipose, and muscle. **Figure** **4**a shows linear relations between *P*_in_ and *P*_+_ for the five *H*. The measured data for *H* = 36.46 and 52.86 mm collapsed to each other. This finding was in agreement with the maximum nominal propagation distance of the applicator, 40 mm. Figure 4b shows that, for different *P*_in_, *P*_+_ is inversely proportional to *H* approximately.

**Figure 5:**
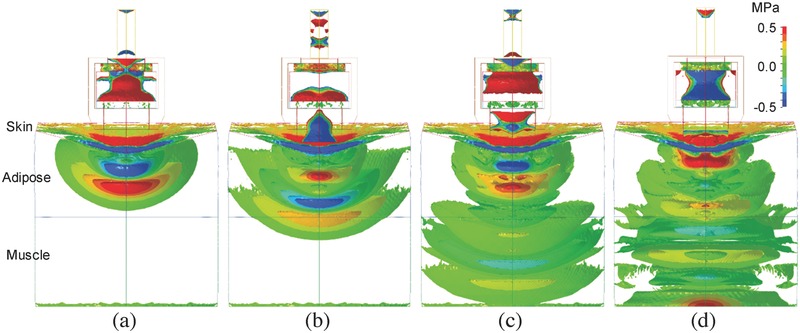
3D temporal and spatial distribution of the wave pressure in soft tissues for *U* = 10 m s^−1^. a) Pressure wave transmits into soft tissues from the applicators in a compression–rarefication pattern. b) Multiple compression–rarefication patterns appear due to reverberations in the applicator. c) Until the wavefront reaches the bottom boundary, the pressure radiates directionally. d) The pressure wave reflects at the bottom boundary, and the distribution of the pressure wave is flattened.

To obtain further understanding of the pressure propagation in soft tissue, simulations were carried out. **Figure** **5** shows the propagation of the pressure wave represented by isosurfaces. Due to the existing preload, the static pressures at the upper and lower surfaces of the skin were positive and negative, respectively (Figure 5a). After the impact of the projectile and the applicator, compression and rarefaction waves propagated in the soft tissues (Figure 5b). Notably, the pressure field showed small discontinuity when passing through the interface between adipose and muscle. This was due to the small differences in acoustic impedances of the tissues. For an incident wave traveling in the normal direction of an interface between media 1 and 2, the reflection coefficient, the ratio of the pressures of the reflected wave and the incident wave, is a function of acoustic impedances

(3) $$R\;=\;\frac{r_{2}\;-\;r_{1}}{r_{2}\;+\;r_{1}}$$

where *r*_1_ and *r*_2_ are acoustic impedances of media 1 and 2, respectively. The transmission coefficient, the ratio of the pressures of the transmitted wave and the incident wave, is

(4) $$T\;=\;\frac{2r_{2}{}_{}}{r_{2}\;+\;r_{1}}\;=\;1\;+\;R$$

The acoustic impedance is the product of density ρ and sound speed *c*. According to typical acoustic properties of tissues,^26^ *R* at the interface between adipose and muscle was 0.10. Correspondingly, *T* at the interface was 1.10. This explained that the discontinuity of the pressure at the interface was small. We also found that *R* and *T* at the interface between skin and adipose were −0.18 and 0.82, respectively. The pressure thus reduced nearly 20% when passing the interface between the skin and adipose.

As shown in Figure 5c, the distribution of the pressure remained a radial pattern until wavefronts reached the bottom boundary. After that, the shape of the isosurfaces became flat (Figure 5d) due to the complete reflection of the incident wave at the bottom boundary that is fixed in simulations. In clinical practices, the lower boundary of muscle was bone. At the interface between muscle and bone, *R* and *T* were 0.64 and 1.64, respectively. The reflection at this interface was thus large enough to influence pressure evolutions. rESWT has been proven to be effective to induce new bone formation.^27^ Without considering the reflection, the pressure on bone surface could be underestimated, as well as the effective areas. When treating muscular disorders, clinicians might need to consider the reflection carefully to avoid any unwanted osteogenesis. It is suggested that the reflection should also be considered during clinical treatments involving bone tissue, for example, nonunion of fracture.

**Figure 6:**
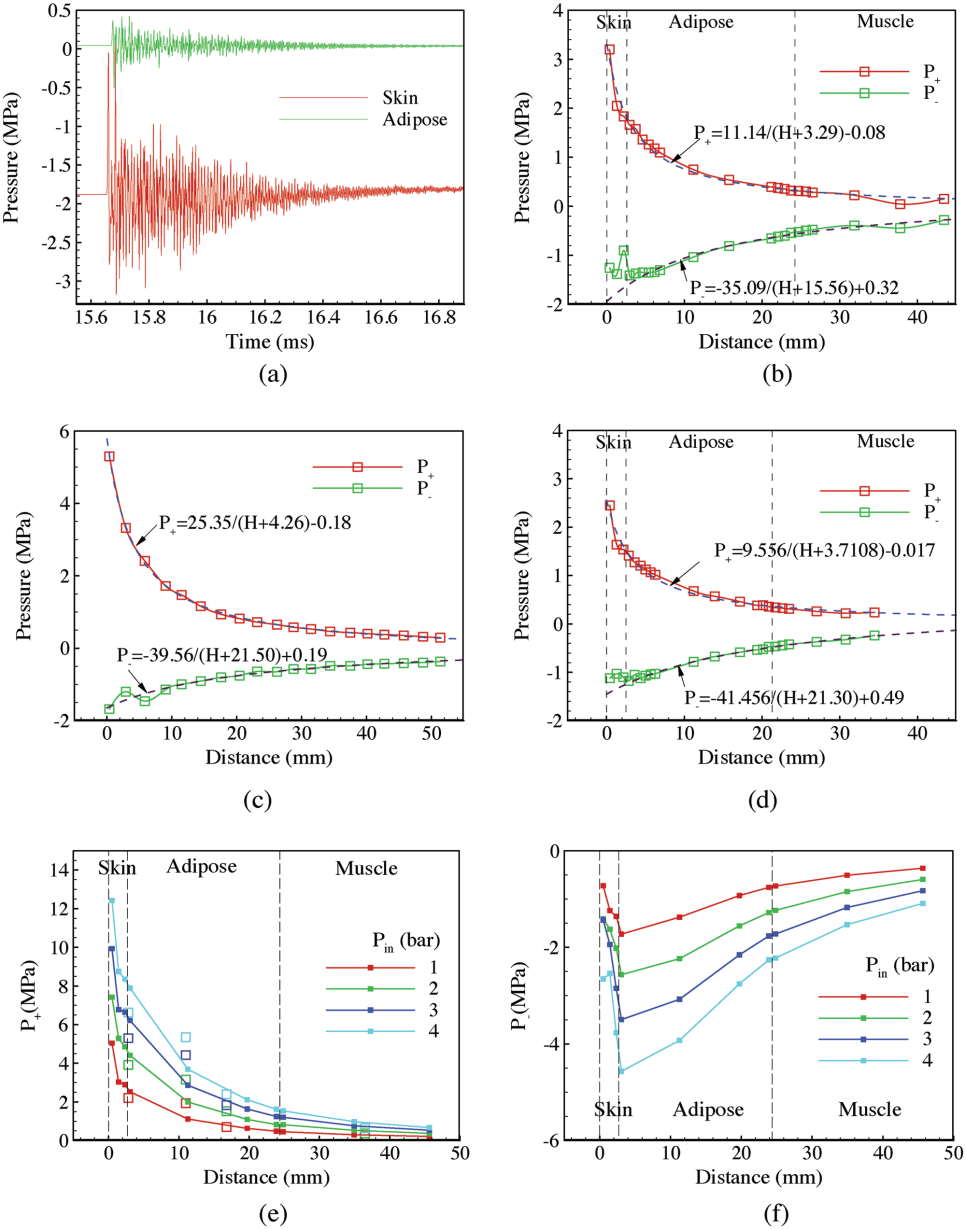
a) Pressure evolutions in skin and adipose for *U* = 10 m s^−1^. b) Variations of *P*_+_ and *P*_−_ along the axial direction in biological tissues for depth of the indentation *D*_i_ = 5.36 mm. c) Variations of *P*_+_ and *P*_−_ along the axial direction in water. d) Variations of *P*_+_ and *P*_−_ along the axial direction in biological tissues for *D*_i_ = 9.11 mm. Variations of e) *P*_+_ and f) *P*_−_ along the axial direction in biological tissues for different *P*_in_.

The evolution of pressure waves in a period larger than 1 ms was recorded with a sampling interval of 0.5 µm. **Figure** **6**a shows waveforms inside the skin and adipose tissues for *U* = 10 m s^−1^. The reverberations in the applicator generated some positive and negative pulses inside the tissues, while the amplitudes decayed exponentially. The overall waveform was similar to measurements in cadaveric experiments by a hydrophone.^19^ Figure 6b shows the axial distribution of *P*_+_ and *P*_−_ of the first compression and rarefaction waves. The amplitudes of *P*_+_ and *P*_−_ were approximately inversely proportional to *H*, except that the variation of *P*_−_ showed complex variation in the skin region. Based on the assumption that the wave can be regarded to be generated by a circular piston, *P*_+_ and *P*_−_ along the axial direction were fitted by

(5) $$P_{+/-}=\;a/(H\;+\;b)\;+\;c$$

as shown in Figure 6b. The values of *c* were small compared to the amplitudes of *P*_+_ and *P*_−_ indicating that the inversely proportional relations were well satisfied. The values of *b* represented the virtual locations of the wave source, which were inside the applicator.

For comparison, we increased the diameter and depth of the water bulk in the petri dish model to simulate the pressure propagation in water with a similar geometry of the soft tissues. Figure 6c shows that the axial variations of *P*_+_ and *P*_−_ in water can be fitted more perfectly with the inversely proportional relation than that in soft tissues. Figure 6b,c shows that the values of *P*_+_ in soft tissues were about 50% of those in water for the same depth. This observation was in line with a previous study,^28^ where the measured pressure within a pig had a peak pressure of about 70% of that in water.

The preload stage caused an indentation in the soft tissue to obtain a full contact between the applicator and the skin. As the depth of the indentation *D*_i_ varied with the force that a therapist applied to press the handpiece against the treating area, the applied force actually varied with each individual and situation. It was thus of interest to study the effect of *D*_i_ on the distribution of the pressure wave. In the previous simulations, the head end of the applicator was just able to make a full contact with the skin at *D*_i_ = 5.36 mm. An additional simulation of *D*_i_ = 9.11 mm was carried out for comparison. As shown in Figure 6d, the absolute values of *P*_+_ and *P*_−_ were smaller than those of *D*_i_ = 5.36 mm in Figure 6b at axial locations. Variation of *D*_i_ also caused changes in tissue thicknesses. The variation of skin thickness was found to be negligible, while the decrease of adipose thickness was 2.83 mm, which accounted for about 75% of the increase of *D*_i_. As a result, *P*_+_ at the interface of adipose and muscle tissues increased about 10.6%. Meanwhile, the absolute value of *P*_−_ decreased about 11.7% with *D*_i_. The effect of *D*_i_ suggested that the preload force could be used to adjust the expected strength that was applied to the target location.

Figure 6e,f shows axial variations of *P*_+_ and *P*_−_ for *P*_in_ varying from 1 to 4 bar at *D*_i_ = 5.36 mm. Figure 6e compares the simulation results of *P*_+_ with experimental measurements. Reasonable agreements between the two results were observed, especially for distances larger than 15 mm. Disparities for distances smaller than 15 mm may be caused by the disparities in mechanical properties of soft tissues. More accurate properties could be obtained by the reverse engineering method to fit tissue mechanical properties using inverse finite element analysis (FEA) of in vivo indentation experiments.^29^ Figure 6f shows that the absolute values of *P*_−_ increased inside the skin and then decreased inside adipose and muscle tissues. The maximum absolute values of *P*_−_ were located at the interface between the skin and adipose tissues. To obtain more information that can guide clinical practices, the data of *P*_+_ and *P*_−_ were fitted by *a*/(*H* + *b*) + *c* for different *P*_in_. The results for *P*_+_ in Table S2 (Supporting Information) showed that both *a* and *b* increased with *P*_in_, while *c* decreased with *P*_in_. In addition, *a* and *c* varied near‐linearly with *P*_in_. Variation of *b* was nonlinear indicating that the virtual location of the wave source varied with *P*_in_. Similar variations were observed in Table S2 (Supporting Information) for *P*_−_.

### 2.3 Pressure Field in Soft Tissues around Lumbar Vertebrae

**Figure 7:**
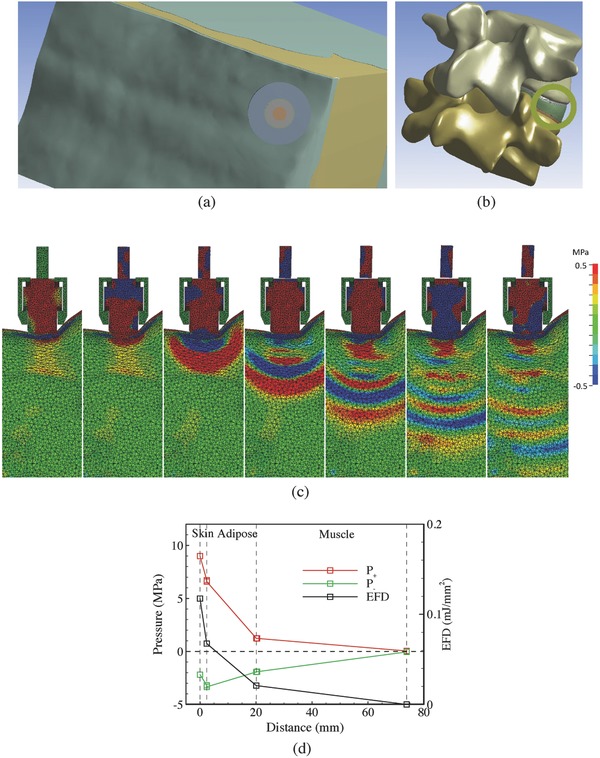
a) The applicator is positioned to the posterolateral position of the back skin. b) The axial direction of the applicator demonstrating by a o‐ring that restricts the applicator. c) Propagation of pressure waves in the soft tissues near the L4/L5 human lumbar vertebrae under *P*_in_ = 4 bar. (The time interval between the snapshots is 10 µs.) d) Variations of *P*_+_, *P*_−_, and EFD along the axial direction in the soft tissues near the L4/L5 human lumbar vertebrae.

After investigating the pressure propagation inside water and porcine tissues, simulations were carried out for a much more complicated situation with realistic geometries of human L4/L5 lumbar vertebrae and surrounding tissues. The preliminary aim was to find whether an effective pressure wave can arrive at the lumbar intervertebral disc. The first step was to find the proper position and direction to place the applicator. The distance between the applicator and the intervertebral disc should be as short as possible, while pressure waves should not be impeded by the lumbar. **Figure** **7**a,b shows such a direction pointing to the lumbar intervertebral disc from the posterolateral position of the back skin. The distance from the skin and the disc was about 74 mm, which was the shortest distance we could achieve anatomically.

The impact velocity of the projectile was set to be 23.16 m s^−1^ to model the treatment of *P*_in_ = 4 bar. Following a similar procedure for the porcine model, a preload displacement was applied to the casing to make the head end of the applicator contact with the skin completely. Figure 7c shows the propagation of compression and rarefaction waves at the cross section along the axial of the handpiece. As the axis of the applicator was not normal to the skin, the applicator has a small degree of tilting after the preload stage. The collision between surfaces of the projectile and the applicator was thus not completely head‐on. This practical factor reduced the amplitude of the pressure wave. Also, the projectile rotated after the impact. In practice, the rotation might cause interaction between the projectile and the guiding tube inside the headpiece to cause wear.

Figure 7d shows the axial distribution of *P*_+_ and *P*_−_ for *P*_in_ = 4 bar, along with the energy flux density (EFD), which is defined as

(6) $$\mathrm{EFD}\;=\;\frac{1}{\rho c_{s}}\int P^{2}dt$$

Despite the similarity in variations along the axial direction, the absolute values of *P*_+_ and *P*_−_ were smaller than those in Figure 6e,f for *P*_in_ = 4 bar. This disparity was likely due to small tilting of the applicator as mentioned above. The degree of indentation may also play a role. The maximum values of *P*_+_ and *P*_−_ at the surface of the intervertebral disc were found to be about 0.045 and −0.047 MPa, respectively, which were most probably too small to generate effective biological effects. For this specific situation, EFD of the waves reduced about 42.5% when the waves propagated across the skin. Only about 17.8% EFD was transmitted from the skin to the interface between adipose and muscle tissues.

## 3 Conclusions and Remarks

In this paper, we have established a combined experimental and numerical methodology to quantify pressure waves generated by a ballistic rESWT device. Direct measurements were carried out by placing a thin and flexible film sensor on the bottom of a petri dish and inside biological tissues. Numerical simulations were based on explicit dynamic analyses considering realistic geometrical and mechanical properties. 3D evolutions of pressure waves were obtained for three configurations, as well as axial and radial distributions of peak pressures. Simulation results were compared with experimental measurements to calibrate and validate numerical models. Pressure waves can be regarded to be generated from a circle piston with a directional distribution of wave energy. The amplitudes of wave pressures along the axial direction are inversely proportional to the axial distance approximately. Finally, numerical simulations were carried out for a more realistic clinical practice, treatment of protrusion of intervertebral disc. Proper applied position and direction were identified from reconstructed tissues based on CT images. Simulations suggested that the pressure wave can arrive at the intervertebral disc. However, the amplitudes were most probably too small to generate effective biological responses. This study combined practicable technologies of pressure measures and simulations. Thin and flexible film sensors were robust for measuring wave pressure inside biological tissues. CT‐based reconstruction of human tissues and explicit dynamics analysis were useful tools to evaluate wave pressures in the target sites. Combined with clinical practices, the methodology can provide a route toward personalized treatment planning and mechanism study for rESWT.

## 4 Modeling Section

Numerical simulations are capable of obtaining comprehensive information of the pressure field, which is difficult for experimental measurements, especially inside biological tissues with complex geometries.[[qv: 12b,30]] Finite element method has been used to simulate the generation and propagation of the ballistic type rESW.^18^, ^20^ However, the finite element method has not been widely used in studying rESWT. One reason is the lack of calibration between the impact velocity of the projectile *U* and the driven pressure *P*_in_ of the rESWT device. Another reason is that numerical and material models have not been validated by experiments. We thus carried out detailed finite element simulations with calibration and validation from experimental measurements.

**Figure 8:**
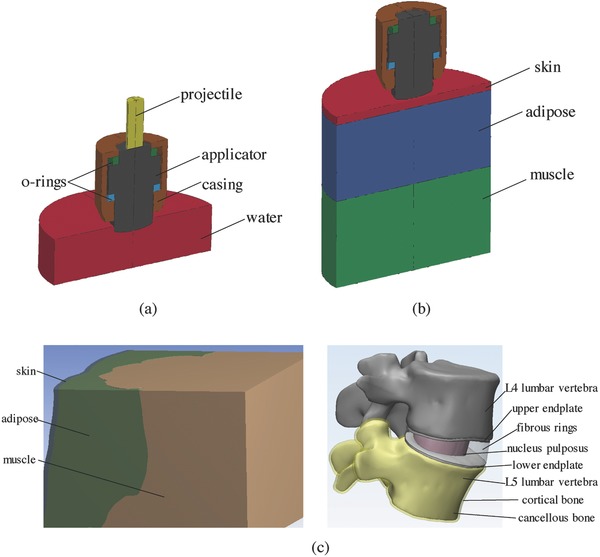
Finite element models. a) Front of the handpiece of an rESWT device (including applicator, projectile, o‐rings, and casing) and water in a petri dish. b) Front of the handpiece of an rESWT device and soft tissues (skin, adipose, and muscle). c) L4/L5 lumbar vertebrae (including cortical and cancellous bone), intervertebral disc (including upper and lower endplate, fibrous rings, and nucleus pulposus), and the surrounding tissues (skin, adipose, and muscle).

The front portion of the handpiece of the rESWT device was constructed by measuring geometrical parameters of the components with permission of the manufacturer, including the projectile, the applicator, two o‐rings, and the casing. Three finite element models were constructed for simulating pressure propagations in water, porcine tissues, and human tissues, respectively. The first one was a water bulk with the shape of the petri dish (**Figure** **8**a). The head end of the applicator was immersed in water. The second one was a three‐layer cylinder with the heights of the skin, adipose, and muscle corresponding to those of the porcine model in experiments (Figure 8b). Only quarters of these two models were simulated due to the axial symmetry, resulting in much reduced computational cost. The third one was the human L4/L5 lumbar and the surrounding tissues that were reconstructed from CT images taken from a healthy volunteer (Figure 8c). The scans were obtained at 1.0 mm intervals using a TOSHIBA/Aquilion CT device (at Beijing Jishuitan Hospital, China). The segmentation was performed using Mimics Research (Materialise NV, Belgium) to reconstruct different tissues as separate entities. After the representation of the vertebra's outer surfaces, the surfaces were offset inward to create a shell with a thickness of 1.0 mm, corresponding to the cortical bone. The remaining volume of each vertebra was considered as cancellous bone. The whole volume of the intervertebral discs of the lumbar spine was reverse engineered based on the superior and inferior surface of the connecting vertebras. The inner nucleus pulposus, upper and lower endplates were generated based on anatomic data. The remaining volume of the intervertebral discs represented the outer annulus. Skin, adipose, and muscle tissues were reconstructed from CT images with proper smoothing operations.

To accurately model the reflection and transmission of displacements and stresses across the interfaces between the tissues, rescaling and Boolean operations were performed using the ANSYS Workbench Design Modeler (ANSYS Inc., USA) to ensure perfect adjoining of the tissues. Note that the tissues were grouped as a multibodied part to achieve joined tetrahedral meshes in the subsequent meshing procedure, and thus no tied contacts are required to be specified. The generation and propagation of the pressure wave were modeled by explicit dynamics simulations. As a typical requirement of explicit dynamic simulation, the models were meshed with uniform constant stress four‐node tetrahedral elements in ANSYS Workbench Mechanical (ANSYS Inc., USA) using a patch‐independent meshing method.

Table S3 (Supporting Information) lists the material properties used for numerical simulations. The projectile and applicator were made of steel and modeled by a linear elastic model. The casing was modeled as a rigid body only for the supporting and preload purposes. A hyperelastic rubber model was used for the o‐rings.^31^ Water was modeled by a shock equation‐of‐state Grüneisen model. A previous study emphasized that the correct simulation of the behavior of biological tissues requires accurate material models considering viscoelasticity.^18^ The soft tissues were thus treated as single‐term Ogden rubber material with quasilinear viscoelastic Prony series for viscoelasticity.^32^ Note that predictive and patient specific biomechanical models can be evaluated using inverse FEA of in vivo indentation experiments.[[qv: 29a]] The other components were modeled as linear elastic materials.

Numerical simulations were performed using LS‐DYNA (Livermore Software Technology Corp., USA), which is a general‐purpose finite element code for analyzing the large deformation static and dynamic response of structures. LS‐DYNA's main solution methodology is based on explicit time integration.^33^ For the simulations of pressure propagations in a petri dish, the bottom and side walls of the water domain were fixed. The normal velocities were set to be zero at the two symmetrical boundaries to model the symmetry. The casing was fixed and an initial velocity *U* was defined for the projectile to mimic the wave generation mechanism in the ballistic type rESWT device. Different from water, soft tissues deform when the applicator is pressed onto the skin before the ESWT treatment. Therefore, an additional preload step was required to achieve good contact before the generation of the pressure waves. The preload step was realized by applying a displacement to the casting until the front of the applicator was fully contacted with the skin. The casing was restricted to move only in the axial direction without any rotations. To reduce the computational time, mass damping was also added to the components except for the projectile and the casing to wipe out the dynamic responses. After the displacement of the casting was complete, simulations kept running until deformations of the components reached steady states. Figure S3a,b (Supporting Information) shows the simulation model before and after the preload step. The damping was then removed, and the simulation resumed for the impact step by defining an initial velocity *U* for the projectile. Note that a simulation without damping was first carried out to determine the optimized damping coefficient, according to the dynamic responses of the soft tissues. All simulations were performed on a 12 CPUs Workstation (HP Z800, Hewlett‐Packard Inc., USA). The preload step took ≈24 h while the impact step took ≈2 h.

## 5 Experimental Section

Experiments were designed to measure the pressure propagation either in the water within a petri dish or inside porcine tissues. Pressure waves were generated by a ballistic shock wave therapy device with a standard 15 mm applicator (Masterpuls MP100, STORZ Medical AG, Switzerland), which created radial shock waves in the medium contacting with the applicator. The collision of the projectile and the applicator caused movement of the headpiece during clinical treatment. To avoid the uncertainty caused by this motion, the handpiece of the ESWT device was firmly clamped by three circular rings of a supporting frame (Figure S4a, Supporting Information). The vertical distance between the tip of the applicator and the measuring position was tuned through the vertical positioning of the rings. This adjustable feature also allowed controlling the contact between the applicator and the porcine skin.

Pressures were measured by a laminar pressure sensor (Jinzhou Kexin Electronic Material Co., Ltd, China) placed at the bottom of a petri dish and inside porcine tissues. The sensor, with an active sensing area of 5 mm in diameter, used a piezoelectric polyvinylidene fluoride (PVDF) film as the sensing element. PVDF has an excellent acoustic impedance match with human tissue as well as water.^34^ The flexibility of the PVDF film sensor made it more suitable to measure the pressure inside biological tissues in vivo^28^ or in vitro,^18^ compared to the needle‐type hydrophone that was easily damaged if exposed to any physical force.[[qv: 16b,19]] To avoid the short circuit of the sensor in the aqueous environment, the sensor was coated with a transparent adhesive to form a thin and soft water‐resisting layer. The sensor was connected to a broadband charge amplifier (YE5856, Sinocera Piezotronics, Inc., China) and a digital phosphor oscilloscope (DPO3054, Tektronix, Inc., USA) to record the pressure data.

To measure the pressure at the bottom of the petri dish, a PVDF sensor was adhered to the center of the petri dish by a cyanoacrylate‐based instant adhesive (Loctite 495, Kreglinger‐Loctite, Belgium). The petri dish of 60 mm in diameter was placed into a groove in an acrylic plate and clamped by tablet clips (Figure S4b, Supporting Information). The acrylic plate was fastened to an *XY* axis crossed roller guide stage (B21‐100CR, Suruga Seiki Co., Ltd., Japan), which was used for alignment of the applicator and the sensor. After the handpiece was adjusted to the desired position, deionized water was added to the petri dish by a syringe slowly until the water surface was just above the cambered head end of the applicator.

Pig is commonly considered as an excellent animal model in many fields of biomedical research.^35^ The PVDF sensor was used to directly measure the pressure inside the porcine tissues. To model the practice situation for treatments of musculoskeletal disorders, a whole porcine portion with several lumbar vertebras and nearby soft tissues was used. The portion was obtained from a local slaughterhouse within 24 h after the pig was slaughtered. The tissues were cut by a surgical knife to create openings perpendicular to the axis of the applicator. The PVDF sensor was wiped throughout with a medical ultrasonic coupling gel and then inserted into the openings by flathead tweezers (Figure S4c, Supporting Information). Approval related to the utilization of porcine tissues and experimental protocols was obtained from the Beijing Jishuitan Hospital Institutional Review Board.

## Conflict of Interest

The authors declare no conflict of interest.

## Supporting information

Supplementary
