# Supplementary material for: Quantitative Assessments of Mechanical Responses upon Radial Extracorporeal Shock Wave Therapy
Source: Adv Sci (Weinh). 2017 Dec 19;5(3):1700797. doi: 10.1002/advs.201700797 (PMC5867036; doi:10.1002/advs.201700797)
Supplement: Supplementary file 1 — Supplementary [file ADVS-5-1700797-s001.pdf]

Copyright WILEY-VCH Verlag GmbH & Co. KGaA, 69469 Weinheim, Germany, 2017.

ADVANCED SCIENCE Open Access

# Supporting Information

for *Adv. Sci.,* DOI: 10.1002/advs.201700797

# Quantitative Assessments of Mechanical Responses upon Radial Extracorporeal Shock Wave Therapy

*Yajun Liu, Xiaodong Chen,* Anyi Guo, Sijin Liu, and Guoqing Hu*

DOI: 10.1002/ advs.201700797

Supporting Information

**Quantitative Assessments of Mechanical Responses upon Radial Extracorporeal Shock Wave Therapy**

*Yajun Liu, Anyi Guo, Xiaodong Chen*, Sijin Liu, and Guoqing Hu*

Dr. Y. Liu, A. Guo
Orthopedic Shock Wave Treatment Center, Spine Surgery Department, Beijing Jishuitan Hospital, Beijing 100035, China
Dr. X. Chen, Prof. G. Hu
The State Key Laboratory of Nonlinear Mechanics, Beijing Key Laboratory of Engineered Construction and Mechanobiology, Institute of Mechanics, Chinese Academy of Sciences, Beijing 100190, China
School of Engineering Science, University of Chinese Academy of Sciences, Beijing 100049, China
E-mail: chenxiaodong@imech.ac.cn
Prof. S. Liu
The State Key Laboratory of Environmental Chemistry and Ecotoxicology, Research Center for Eco-Environmental Sciences, Chinese Academy of Sciences, Beijing 100085, China

Keywords: extracorporeal shock wave therapy, experimental measurement, explicit dynamics analysis

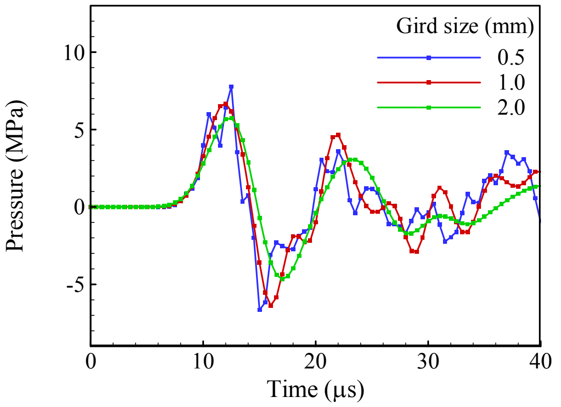


**Figure S1.** Effect of grid resolutions on the pressure evolution at the bottom center of the petri dish at $H$ = 5 mm and $U$ = 10 m/s. The differences between $\Delta$ = 0.5 and 1.0 were smaller than those between $\Delta$ = 1.0 and 2.0, suggesting the convergence of the mesh.

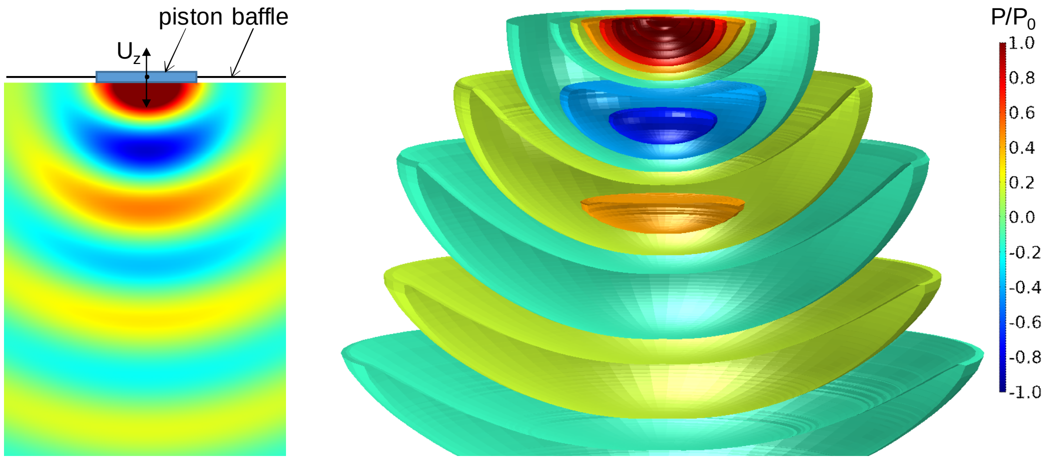


(a) (b)

**Figure S2.** (a) Schematic diagram of a vibrating circular piston set in a large baffle. The piston vibrates with a velocity of $U_z$. The pressure contour shows the wave pressure at the downstream of the piston at a frequency of 90,909 Hz (the period of vibration is about 11 $\mu$s). (b) Three-dimensional representation of the pressure field (pressure amplitude $P_0$ = 1 MPa). The radiation of the rESWT in Figure 2 was similar to that in (b).

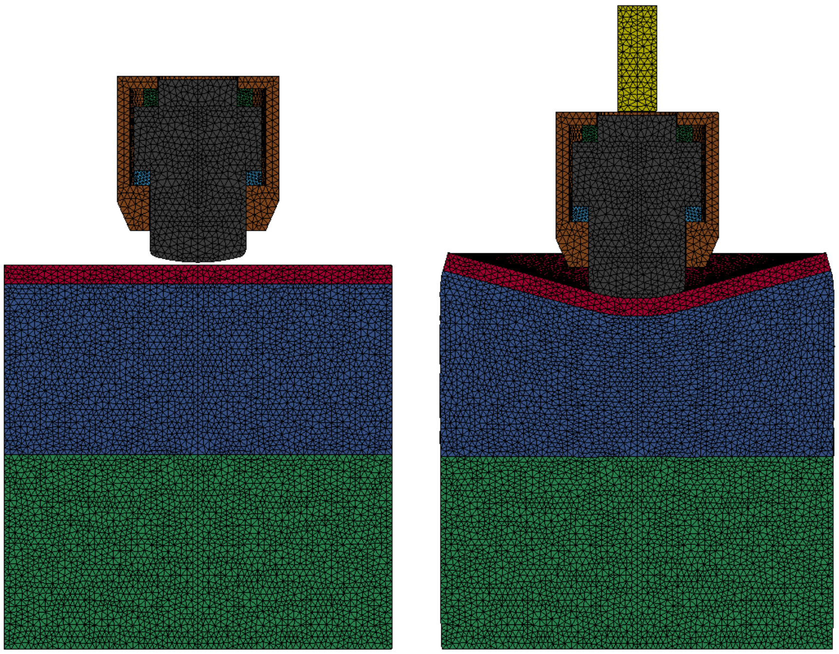

(a) (b)

**Figure S3.** Finite element meshes before (a) and after (b) the preload step. Before the preload step, there was a distance between the head-end of the applicator and the skin surface. A displacement was applied to the casing gradually until the front surface of the applicator contacted the skin completely. The casing thus stayed still to provide compression of the soft tissues.

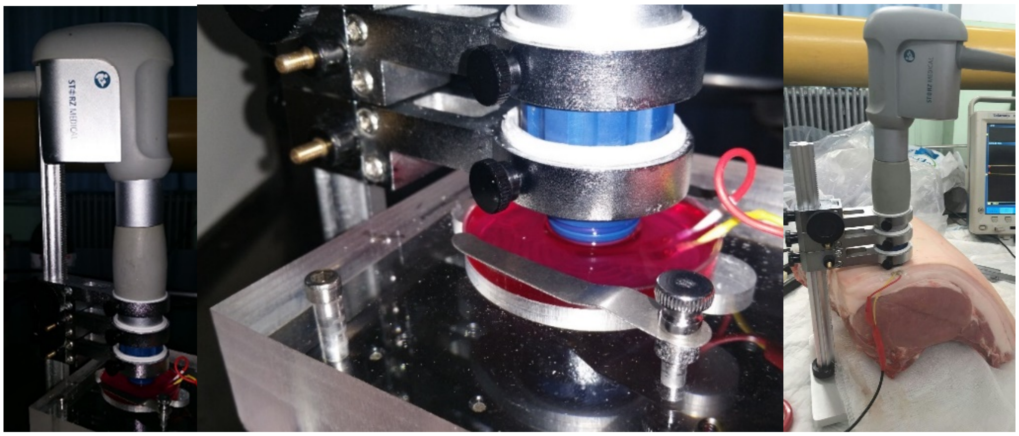

(a) (b) (c)

**Figure S4.** Experimental apparatus for measuring of pressure either at the bottom of a petri dish or inside porcine tissues. (a) A supporting frame holding the handpiece of the ESWT device vertically; (b) Setup for measuring pressure at the bottom of a petri dish; (c) Setup for measuring the pressure inside porcine tissues.

**Table S1.** Depths *H* of measured locations and the corresponding tissue thicknesses.

| | *H* (mm) | | | |
|---|---|---|---|---|
| Position | Skin | Adipose | Muscle | Total |
| 1 | 2.82 | 0 | 0 | 2.82 |
| 2 | 2.82 | 8.18 | 0 | 11.00 |
| 3 | 2.82 | 13.96 | 0 | 16.78 |
| 4 | 2.82 | 26.50 | 7.14 | 36.46 |
| 5 | 2.82 | 26.50 | 23.54 | 52.86 |

**Table S2.** Fitting parameters of $P_+$ and $P_-$ along the axial direction for different $P_{in}$.

| $P_{in}$ | $P_+$ | | | $P_-$ | | |
|---|---|---|---|---|---|---|
| bar | *a* (MPa·mm) | *b* (mm) | *c* (MPa) | *a* (MPa·mm) | *b* (mm) | *c* (MPa) |
| 1 | 11.11 | 1.815 | 0.07 | -36.34 | 10.52 | 0.29 |
| 2 | 28.08 | 3.33 | -0.15 | -76.50 | 14.93 | 0.68 |
| 3 | 41.72 | 3.82 | -0.21 | -108.75 | 15.62 | 0.96 |
| 4 | 55.30 | 4.04 | -0.31 | -133.37 | 15.12 | 1.12 |

**Table S3** lists the material properties used for numerical simulations. The projectile and applicator were made of steel and modeled by the linear elastic model with Young's modulus *E* and Poisson's ratio *v*. The casing was modeled as a rigid body only for the supporting and preload purposes. A hyperelastic rubber model (three constant *v*, $C_{10}$ and, $C_{01}$) was used for the o-rings.[1] Water was modeled by a shock equation of state (EOS) Gruneisen model (two constant C and, $S_1$).[2] A previous study emphasized that the correct simulation of the

behavior of biological tissues requires accurate material models considering viscoelasticity.[3] The soft tissues were treated using the single-term Ogden rubber model with quasi-linear viscoelastic Prony series for viscoelasticity.[4] Note that predictive and patient specific biomechanical models can be evaluated using inverse finite element analysis (FEA) of *in-vivo* indentation experiments.[5] The other components were modelled as linear elastic materials.

**Table S3**. Material properties of device components, water, and biological tissues.

| | Model | Parameters |
|---|---|---|
| Projectile | Linear elastic | $\rho$ = 7800 kg/m$^3$, $E$ = 2.0 × 10$^{11}$Pa, $v$ = 0.3 |
| Applicator | | |
| Casing | Rigid | $\rho$ = 7800 kg/m$^3$ |
| O-ring | Hyperelastic rubber | $\rho$ = 1150 kg/m$^3$, $v$ = 0.4988, $C_{10}$= 1.933×10$^6$ Pa, $C_{01}$= 0.483×10$^6$ Pa [1] |
| Water | Shock EOS Gruneisen | $\rho$ = 998 kg/m$^3$, C = 1647 m/s, $S_1$ = 1.921 [2] |
| Skin | Single-term Ogden with Prony series | $\rho$ = 1110 kg/m$^3$, $\mu$ = 2.20×10$^6$ Pa, $\alpha$= 12, $g$(1) = 5.01×10$^1$ Pa, $\tau$(1) = 5.73×10$^{-1}$ s, $g$(2) = 4.44×10$^{-1}$ Pa, $\tau$(2) = 9.47 s [4] |
| Adipose | | $\rho$ = 1100 kg/m$^3$, $\mu$ = 1.70×10$^3$ Pa, $\alpha$ = 23, $g$(1) = 1.59×10$^{-2}$ Pa, $\tau$(1) = 7.83×10$^{-5}$ s, $g$(2) = -7.97×10$^{-2}$ Pa, $\tau$(2) = 1.17×10$^{-3}$ s, $g$(3) = -5.89×10$^{-1}$ Pa, $\tau$(3) = 1.61 s, $g$(4) = 1.25×10$^{-1}$ Pa, $\tau$(4) = 7.29×10$^1$ s [4] |
| Muscle | | $\rho$ = 920 kg/m$^3$, $\mu$ = 3.63×10$^4$ Pa, $\alpha$ = 45, $g$(1) = 3.30×10$^{-1}$ Pa, $\tau$(1) = 2.37 s, $g$(2) = 2.56×10$^{-1}$ Pa, $\tau$(2) = 7.02×10$^1$ s [4] |
| Cortical bone | Linear elastic | $\rho$ = 1850 kg/m$^3$, $E$ = 1.2 × 10$^{10}$Pa, $v$ = 0.3 |
| Cancellous bone | Linear elastic | $\rho$ = 250 kg/m$^3$, $E$ = 1.06 × 10$^8$Pa, $v$ = 0.2 |
| Nucleus pulposus | Linear elastic | $\rho$ = 1000 kg/m$^3$, $E$ = 1.0 × 10$^6$Pa, $v$ = 0.499 |
| Fibrous rings | Linear elastic | $\rho$ = 1000 kg/m$^3$, $E$ = 2.95 × 10$^8$Pa, $v$ = 0.35 |
| Endplates | Linear elastic | $\rho$ = 1000 kg/m$^3$, $E$ = 2.4 × 10$^7$Pa, $v$ = 0.4 |

where, $\rho$ is density; $E$ is Young's modulus; $v$ is Poisson's ratio. $C_{10}$ and $C_{01}$ are constants in hyperelastic rubber model; C and, $S_1$ are constants in shock equation of state (EOS) Gruneisen model; $\mu$ and $\alpha$ are constants in Ogden rubber model; $g$(i) and $\tau$(i) are quasi-linear viscoelastic Prony series for viscoelasticity.

**References**


[1] C. Liao, W. Huang, Y. Wang, S. Suo, Y. Liu, *Chin. J. Mech. Eng.* **2013**, 26, 85.

[2] Group GMX-6, Los Alamos Scientific Laboratory, 1969.

[3] M. Benoit, J. H. Giovanola, A. Curnier, K. Agbeviade, M. Donnet, presented at DYMAT-International Conference on the Mechanical and Physical Behaviour of Materials under Dynamic Loading **2009**.

[4] T. Payne, S. Mitchell, R. Bibb, M. Waters, *J. Mech. Behav. Biomed. Mater.* **2015**, 41, 336.

[5] D. M. Sengeh, K. M. Moerman, A. Petron, H. Herr, *J. Mech. Behav. Biomed. Mater.* **2016**, 59, 379.
